# Phase I/II Study of Neoadjuvant Chemoradiotherapy Consisting of S-1 and Cisplatin for Patients with Clinically Resectable Type 4 or Large Type 3 Gastric Cancer (OGSG1205)

**DOI:** 10.1245/s10434-024-16845-x

**Published:** 2025-01-15

**Authors:** Masayuki Shinkai, Motohiro Imano, Masaki Yokokawa, Ryo Tanaka, Jin Matsuyama, Toshio Shimokawa, Hisato Kawakami, Taroh Satoh, Takushi Yasuda, Hiroshi Furukawa

**Affiliations:** 1 Department of Surgery, Faculty of Medicine, Kindai University, Osaka-Sayama, Osaka Japan; 2 Department of Radiation Oncology, Faculty of Medicine, Kindai University, Osaka-Sayama, Osaka Japan; 3 Department of General and Gastroenterological Surgery, Osaka Medical and Pharmaceutical University, Takatsuki, Osaka Japan; 4 Department of Gastroenterological Surgery, Higashiosaka City Medical Center, Higashiosaka, Osaka Japan; 5 Clinical Study Support Center, Wakayama Medical University, Wakayama, Japan; 6 Department of Medical Oncology, Faculty of Medicine, Kindai University, Osaka-Sayama, Osaka Japan; 7 Center for Cancer Genomics and Precision Medicine, Osaka University Hospital, Suita, Osaka Japan

**Keywords:** Gastric cancer, Chemoradiotherapy, Type 4, Large type 3, S-1, Cisplatin, Phase I/II

## Abstract

**Background:**

To improve the prognosis of clinically resectable type 4 or large type 3 gastric cancer (GC), we performed a phase I/II study of neoadjuvant-radiotherapy combined with S-1 plus cisplatin.

**Patients and Methods:**

Phase I, with a standard 3 + 3 dose-escalation design, was performed to define the recommended phase II dose. Efficacy and safety were evaluated in phase II. The three dose levels were as follows: level 0, S-1 60 mg/m^2^ on days 1–14 plus cisplatin 60 mg/m^2^ on day 1; level 1, S-1 80 mg/m^2^ on days 1–14 plus cisplatin 60 mg/m^2^ on day 1; and level 2, S-1 80 mg/m^2^ on days 1–14 and 22–35, plus cisplatin 60 mg/m^2^ on days 1 and 22. The starting dose was level 1. Radiotherapy was delivered at a total dose of 40 Gy in fractions for 4 weeks.

**Results:**

A total of six patients were enrolled in the phase I study. Dose-limiting toxicity was observed at level 2; level 1 was established as the recommended phase II dose. In phase II, 20 patients were enrolled from November 2012 to April 2018. Grade 3/4 leukopenia and nonhematologic adverse events occurred in 35% and 5% of the patients, respectively. In total, 19 patients underwent the protocol surgery; 2 (10.5%) achieved a pathological complete response. There were no treatment-related deaths; 3- and 5-year overall survival rates were 70.0 and 50.0%, respectively.

**Conclusions:**

Neoadjuvant chemoradiotherapy with S-1 plus cisplatin is a safe and promising treatment for clinically resectable type 4 or large type 3 GC.

Type 4 gastric cancer (GC), according to the Japanese Gastric Cancer Association, indicates Borrmann type IV carcinoma in the American Joint Committee on Cancer (AJCC) staging and National Comprehensive Cancer Network (NCCN) guidelines.^1^ The terms linitis plastica ^2^ and scirrhous carcinoma^3^ are also used in English publications to refer to type 4 GC.

Type 4 GC has a poorer prognosis than that of other GC types.^4,5^ The standard treatment for resectable type 4 GC is radical gastrectomy, the outcomes of which remain unsatisfactory. The 5-year survival rate after gastrectomy is approximately 30%.^6^

To improve the poor prognosis with type 4 GC, neoadjuvant chemotherapy (NAC) was hypothesized to be preferable to eradicate micrometastases and achieve higher compliance with intensive chemotherapy. On the basis of this hypothesis, the Japan Clinical Oncology Group (JCOG) performed a phase III study (JCOG0501) to confirm the superiority of neoadjuvant S-1 plus cisplatin followed by extended systemic lymphadenectomy (D2) gastrectomy over upfront surgery, primarily in patients with type 4 GC. Although the curative resection rates were 66.6% in the upfront surgery group and 80.6% in the neoadjuvant group, the 3-year overall survival rates were 62.4% and 60.9%, respectively. The JCOG0501 trial did not show a survival benefit of NAC for advanced GC.^7^ Thus, a new strategy is needed to improve the outcomes of type 4 GC.

Saikawa et al. investigated the efficacy of chemoradiotherapy (CRT) with S-1 plus low-dose cisplatin for unresectable GC and reported a high response rate (65.5%).^8^ Additionally, a phase I study of neoadjuvant CRT consisting of S-1 and low-dose cisplatin for patients with resectable advanced GC has been performed. In this phase I study, there were no major surgical complications, and a pathological complete response rate of 10% was reported.^9^ These outcomes indicate the possibility of CRT with S-1 and cisplatin as a new treatment for advanced GC. Therefore, we, the Osaka Gastrointestinal Cancer Chemotherapy Study Group (OGSG), developed a new regimen that involved concurrent radiotherapy and a systemic chemotherapy regimen with S-1 and bolus cisplatin for patients with advanced GC. In this study, we enrolled patients with large type 3 GC in addition to patients with type 4 GC, because tumor size is associated with the recurrence rate.^10^ According to a previous report, the biological characteristics of type 3 GC with a tumor diameter ≥ 8 cm are similar to those of type 4 GC, such as the high incidence of peritoneal dissemination.^11^

In this multicenter phase I/II study, we evaluated the efficacy and safety of neoadjuvant-radiotherapy combined with S-1 plus cisplatin for clinically resectable type 4 GC or large type 3 GC.

## Patients and Methods

### Patients

The specifics of the OGSG1205 trial have been published.^12^ The eligibility criteria for the present study were as follows: (1) histologically proven and clinically resectable GC; (2) age 20–75 years; (3) macroscopic type of carcinoma as type 4 or type 3 GC; (4) in type 3 GC, the required tumor diameter was ≥ 8 cm; (5) Eastern Cooperative Oncology Group performance status of 0 or 1; (6) tumor invasion of the esophagus ≤ 1 cm, with no involvement of the duodenum; (7) lymph node metastasis limited to the regional lymph nodes; (8) no evidence of distant metastases, no peritoneal metastasis, and negative lavage cytology confirmed by staging laparoscopy; (9) no prior abdominal surgery; (10) no previous chemotherapy or radiotherapy; (11) no other previous or concurrent malignancies; (12) no bleeding from the main lesion or intestinal stenosis; and (13) adequate bone marrow function (white blood cell count ≥ 3000/mm^3^, neutrophil count ≥ 1500/mm^3^, hemoglobin ≥ 8.0 g/dL, and platelet count ≥ 100 × 10^3^/mm^3^), adequate liver function (total serum bilirubin level ≤ 2.0 mg/dL and serum alanine transaminase and aspartate transaminase ≤ 100 U/L), and adequate renal function (creatinine clearance ≥ 60 mL/min). Written informed consent was obtained from all patients prior to their participation in the study.

The exclusion criteria were as follows: (1) major medical disease or malignancy other than GC; (2) history of severe drug hypersensitivity; (3) pregnancy or breast feeding; (4) treatment with a major tranquilizer, steroids, flucytosine, phenytoin, or warfarin; (5) lung fibrosis, intestinal pneumonitis, bowel obstruction, or ischemic heart disease; and (6) patients determined inappropriate for inclusion in this study.

The protocol was approved by the institutional review and ethics board of each participating hospital and registered in the University Hospital Medical Information Network (UMIN) database (UMIN000008964).

### Study Design

This study was designed as a multi-institutional open-label phase I/II trial. The objective of the phase I study was to evaluate the maximum tolerated dose (MTD) and dose-limiting toxicities (DLTs) to determine the recommended dose (RD) of S-1 plus cisplatin with concurrent radiotherapy. The primary endpoint of the phase I study was the number of patients with DLTs. The secondary endpoint was the pathological complete response rate.

The objective of the phase II study was to evaluate the efficacy and safety of neoadjuvant-radiotherapy combined with S-1 plus cisplatin in patients with type 4 or large type 3 GC. The primary endpoint of the phase II study was the pathological complete response rate in all eligible patients, including the patients who received treatment at the RD level in the phase I study. The secondary endpoints were the pathological response rate, progression-free-survival (PFS), overall survival (OS), operation completion rate, rate of R0 resection, rate of treatment completion, and the rates of postoperative complications and adverse events (AEs).

### Treatment

**Fig. 1:**
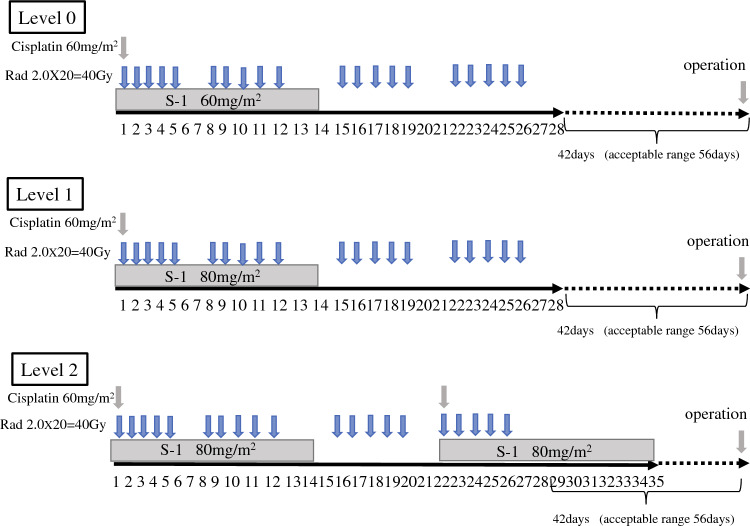
Schema of dose escalation in the phase I study

The treatment schedule is summarized in Fig. 1. Combined CRT consisted of S-1, cisplatin, and radiotherapy. S-1 was administered orally twice per day. In the phase I study, S-1 was administered from days 1 to 14 followed by 14 days of rest at levels 0 and 1. At level 2, S-1 was administered from days 1 to 14 and days 22–35. The dose of S-1 administered at level 0 was 60 mg/m^2^/day. At levels 1 and 2, the dose of S-1 was 80 mg/m^2^/day.^13^ Cisplatin was administered at a dose of 60 mg/m^2^ at levels 0 and 1 on day 1 only. At level 2, cisplatin was administered at a dose of 60 mg/m^2^ on days 1 and 22.

Patients received 2 Gy/day of radiation 5 days per week from the initiation of chemotherapy, and the total radiation dose was 40 Gy. The clinical target volume included the gross volume of the primary tumor and the metastatic lymph nodes plus 1 cm margins that included subclinical extension. We previously reported the details of how to set the irradiation range.^12^

All patients were assessed 4 weeks after the end of CRT by abdominal and pelvic computed tomography to evaluate the possibility of R0 resection.

The surgical criteria were as follows: (1) achievable R0 resection; (2) white blood cell count ≥ 2500/mm^3^; and (3) platelet count ≥ 100,000/mm^3^.

Gastrectomy with en bloc D2 lymph node dissection was performed between 7 weeks and 9 weeks after the end of radiotherapy. Following R0 resection, 1 year of adjuvant chemotherapy with S-1 monotherapy was administered within 6 weeks after gastrectomy.

### Dose Escalation Schedule and DLT

In the phase I study, there were three dose levels for S-1 and cisplatin. Level 1 was the starting dose, and, initially, three patients received this dose. If DLTs developed, an additional three patients were required. Once DLT development was confirmed in 3/6 patients at level 1, the next step comprised level 0. In principle, the RD was one level down from the MTD. However, if the MTD was not expressed at level 2 in this study, we recommended level 2 as the RD.

DLT was defined as follows: (1) grade 4 neutropenia; (2) grade 4 thrombocytopenia; (3) grade 3 febrile neutropenia lasting 4 days; (4) grade 3 nonhematologic toxicity except for appetite loss and general fatigue; and (5) inability to receive S-1 for > 10 days at levels 0 and 1 and > 19 days at level 2.

### Assessment

The tumor-node-metastasis categories were in accordance with the Japanese Classification of Gastric Carcinoma (3rd English edition).^1^ The pathological response rate was evaluated and graded by pathologists in accordance with the Japanese Classification of Gastric Carcinoma (3rd English edition) as grade 0 (no evidence of effect), grade 1a (viable tumor cells remain in more than two-thirds of the tumorous area), grade 1b (viable tumor cells remain in more than one-third but less than two-thirds of the tumorous area), grade 2 (viable tumor cells remain in less than one-third of the tumorous area), or grade 3 (no viable tumor cells). A pathological response was defined as a response greater than grade 1b. Toxicity and AEs were described in accordance with the National Cancer Institute Common Toxicity Criteria version 4.0.^14^ Intra- and postoperative complications were graded in accordance with the Clavien–Dindo classification.^15^

### Follow-up

Relapses were detected by imaging studies, including ultrasonography, computed tomography (CT), and endoscopy. Patients underwent at least one type of imaging study, usually CT, at 6-month intervals until 5 years after surgery.

### Statistical Analysis

The sample size in the phase II study was 25 patients, including those treated at the RD level in the phase I study. This sample size provided 90% power under the hypothesis that the expected pathological complete response rate was 2% and the threshold value was 15% using one-sided testing at a 5% significance level. OS and PFS were calculated from the date of the initial staging laparoscopy to death or the date of the most recent follow-up, respectively. OS and PFS were estimated using the Kaplan–Meier method. All statistical analyses were performed using SAS version 9.2 (SAS Institute Inc., Cary, NC, USA).

## Results

### Phase I Study

Between November 2012 and April 2013, six patients were recruited for the phase I study. The baseline characteristics of these patients are presented in Table 1; three patients were registered at level 1 and no DLT was observed at this dose level. At dose level 2, grade 3 leukopenia and neutropenia were observed in all patients, and grade 3 thrombocytopenia and nausea were observed in one patient each (Table 2). As a result, at dose level 2, DLTs were observed in all patients (inability to receive S-1 for hematological reasons: two patients and grade 3 nausea: one patient). Therefore, the RD was determined as the level 1 dose. The pathological complete response rate, which was the secondary endpoint of the phase I study, was 16.7% (1/6 patients).

**Table 1:** Baseline patient characteristics

Characteristic	Phase I (*n* = 6)	Phase II (*n* = 20)
Age, years		
Median (range)	67 (48–71)	67 (38–74)
Sex		
Male	3	13
Female	3	7
ECOG performance status		
0	6	20
1	0	0
Macroscopic gastric cancer findings (JGCA)		
Type 3	4	10
Type 4	2	10
Tumor location in the stomach		
Upper	0	6
Middle	6	11
Lower	0	3
Histological subtype		
Tubular adenocarcinoma	2	3
Poorly differentiated adenocarcinoma	4	12
Signet-ring cell carcinoma	0	4
Mucinous adenocarcinoma	0	1
Clinical T stage		
T3	0	2
T4a	6	17
T4b	0	1
Clinical N stage		
N0	2	6
N1	4	9
N2	0	5
N3	0	0
Peritoneal metastasis		
P0	6	20
P1	0	0
Peritoneal lavage cytology		
CY0	6	20
CY1	0	0
Distant metastasis		
M0	6	20
M1	0	0
Clinical TNM stage		
IA	0	0
IB	0	0
IIA	0	2
IIB	2	4
IIIA	4	8
IIIB	0	5
IIIC	0	1
IV	0	0

*ECOG* Eastern Cooperative Oncology Group, *JGCA* Japan Gastric Cancer Association,

*P0* no peritoneal metastasis, *P1* peritoneal metastasis, *CY0* peritoneal cytology negative for carcinoma cells, *CY1* peritoneal cytology positive for carcinoma cells, *M0* no distant metastasis, *M1 d*istant metastasis, *TNM* tumor, node, metastasis

**Table 2:** Adverse events in the phase I study (n = 6)

Toxicity	Grade 1	Grade 2	Grade 3	Grade 4	% grade 3/4
Level 1 (*n* = 3)					
Hematologic					
Leukopenia	0	2	1	0	33
Neutropenia	0	2	1	0	33
Thrombocytopenia	2	1	0	0	0
Anemia	1	2	0	0	0
Hypoalbuminemia	1	2	0	0	0
Hyperkalemia	0	0	1	0	33
Hypernatremia	0	1	0	0	0
Gastrointestinal					
Nausea	1	1	0	0	0
Anorexia	2	1	0	0	0
Fatigue	2	1	0	0	0
Malaise	1	1	0	0	0
Level 2 (*n* = 3)					
Hematologic					
Leukopenia	0	0	3	0	100
Neutropenia	0	0	3	0	100
Thrombocytopenia	1	1	1	0	33
Anemia	1	2	0	0	0
Hypercreatininemia	2	0	0	0	0
Hypoalbuminemia	2	1	0	0	0
Hypokalemia	2	0	0	0	0
Hypernatremia	1	0	0	0	0
Gastrointestinal					
Nausea	1	1	1	0	33
Vomiting	0	1	0	0	0
Anorexia	1	2	0	0	0
Fatigue	1	2	0	0	0
Malaise	1	2	0	0	0

Toxicities were graded in accordance with the National Cancer Institute Common Toxicity Criteria for Adverse Events version 4.0.

### Phase II Study

Between November 2012 and April 2018, 20 patients, including the 3 patients in the phase I study who had received the RD of S-1 and cisplatin, were enrolled from three institutions. The baseline characteristics of these patients are presented in Table 1. The median age was 67 years (range 38–74 years). There were ten patients each with large type 3 and type 4 tumors.

### AEs in the Phase II Study

A safety analysis of neoadjuvant CRT was performed in all treated patients. AEs in the phase II study are presented in Table 3. The most common grade 3/4 hematological toxicities were leukopenia (35%) and neutropenia (25%), and grade 4 hyponatremia was observed in one patient (5%). Regarding grade 3/4 nonhematologic toxicities, there was one case each of grade 3 diarrhea and grade 3 febrile neutropenia. There were no treatment-related deaths during neoadjuvant CRT.

**Table 3:** Adverse events in the phase II study (n = 20)

Toxicity	Grade 1	Grade 2	Grade 3	Grade 4	% grade 3/4
Hematologic					
Leukopenia	0	4	6	1	35
Neutropenia	1	4	4	1	25
Thrombocytopenia	5	1	0	0	0
Anemia	6	7	0	0	0
Hypoalbuminemia	2	6	0	0	0
ALT elevation	0	0	1	0	5
AST elevation	1	0	0	0	0
Hyperbilirubinemia	1	0	0	0	0
Hypercreatininemia	3	0	0	0	0
Hyperkalemia	0	0	1	0	5
Hypokalemia	3	0	1	0	5
Hypernatremia	0	1	0	0	0
Hyponatremia	1	0	0	1	5
Gastrointestinal					
Nausea	4	5	0	0	0
Vomiting	2	0	0	0	0
Diarrhea	1	0	1	0	5
Anorexia	4	6	0	0	0
Stomatitis	1	1	0	0	0
Fatigue	5	1	0	0	0
Malaise	7	1	0	0	0
Facial edema	1	0	0	0	0
Febrile neutropenia	–	–	1	0	5

*ALT* alanine transaminase, *AST* aspartate transaminase

Toxicities were graded in accordance with the National Cancer Institute Common Toxicity Criteria for Adverse Events version 4.0.

### Surgery and Postoperative Complications

A total of 19 patients underwent the protocol surgery. The remaining patient was judged ineligible for surgery owing to an AE (grade 4 hyponatremia). The operation completion rate was 95% (19/20 patients). Total gastrectomy was performed in 17 patients, while distal gastrectomy was performed in 2 patients.

In total, 2 of the 19 patients underwent D2 lymph node dissection plus paraaortic lymph node dissection. Peritoneal metastasis (P1) was observed in two patients and one patient had disseminated nodules in the small intestine and underwent partial small bowel resection. The R0 resection rate was 85% (17/20 patients). Other surgical findings are presented in Table 4.

**Table 4:** Surgical findings and postoperative complications

Finding	Phase I (*n* = 6)	Phase II (*n* = 19)
Peritoneal lavage cytology		
CY0	5	17
CY1	1	2
Peritoneal metastasis		
P0	6	17
P1	0	2
Distant metastasis		
M0	4	16
M1	2	3
Type of resection		
Total gastrectomy	6	17
Distal gastrectomy	0	2
Combined resection		
Spleen	6	10
Transverse colon	2	4
Gallbladder	3	6
Pancreatic tail	1	1
Small intestine	0	1
Lymph node dissection		
D2	6	17
D3	0	2
Residual tumor status		
R0	5	17
R1	1	1
R2	0	1
Postoperative complications		
Anastomotic leakage	0	1 (Gr. IIIa)
Pancreatic fistula	0	1 (Gr. IIIa)
Intra-abdominal bleeding	0	1 (Gr. IIIb)
Gastrointestinal bleeding	0	1 (Gr. IIIa)
Portal vein thrombosis	0	1 (Gr. II)
30/60-day mortality	0/0	0/0

*CY0* peritoneal cytology negative for carcinoma cells, *CY1* peritoneal cytology positive for carcinoma cells, *P0* no peritoneal metastasis, *P1* peritoneal metastasis, *M0* no distant metastasis, *M1* distant metastasis, *Gr.* toxicity grade in accordance with the Clavien–Dindo classification

Grade II or higher surgical complications were observed in five patients (26.3%); these comprised anastomotic leakage, pancreatic fistula, intra-abdominal bleeding, gastrointestinal bleeding, and portal vein thrombosis, respectively. In addition, four of the five patients had grade III complications; however, there were no surgery-related deaths.

Although most patients developed pancreatic tissue atrophy postoperatively, none developed diabetes or required pancreatic digestive enzyme replacement therapy.

### Pathological Findings

The pathological effect of neoadjuvant CRT was as follows: grade 0 in 0 (0%) patients, grade 1a in 0 (0%) patients, grade 1b in 6 (31.5%) patients, grade 2 in 11 (58%) patients, and grade 3 in 2 (10.5%) patients. The pathological complete response rate as the primary endpoint in the phase II study was 10.5%, and the pathological response rate was 100% in 19 patients (Table 5).

**Table 5:** Pathological findings

Finding	Phase I (*n* = 6)	Phase II (*n* = 19)
Depth of tumor invasion		
T0	1	2
T1a	0	1
T1b	0	1
T2	1	3
T3	3	9
T4a	1	3
Lymph node metastasis		
N0	4	12
N1	1	2
N2	1	1
N3a	0	4
JCGA stage		
0	0	2
IA	0	2
IB	1	3
IIA	1	3
IIB	1	3
IIIA	1	1
IIIB	0	2
IIIC	0	0
IV	2	3
JCGA histological response (primary tumor)		
Grade 0	0	0
Grade 1a	0	0
Grade 1b	1	6
Grade 2	4	11
Grade 3	1	2

*JCGA* Japanese Classification of Gastric Carcinoma (3rd English edition)

### Postoperative Chemotherapy

S-1 postoperative adjuvant chemotherapy was initiated in 17 patients who underwent R0 resection. Postoperative adjuvant chemotherapy was started an average of 39 days after surgery (range 20–72 days). The AEs associated with postoperative chemotherapy were relatively mild, and there were no grade 4 toxicities throughout the treatment period. Consequently, the completion rate of the protocol treatment comprising neoadjuvant CRT, surgical resection, and postoperative S-1 was 85% (17/20 patients).

### Survival

**Fig. 2:**
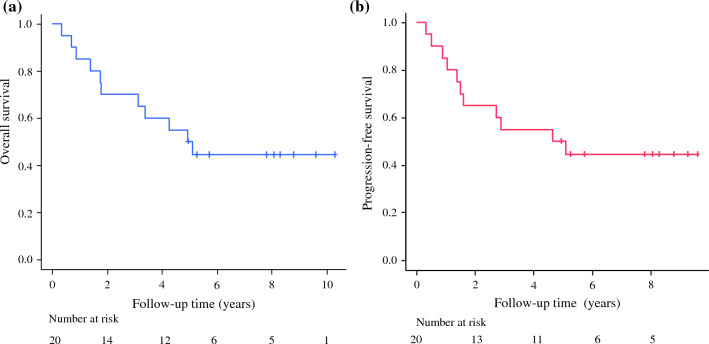
Kaplan–Meier analyses of (**a**) overall survival and (**b**) progression-free survival for the 20 eligible patients

OS and PFS were examined in the 20 eligible patients. The median follow-up period was 60.2 months. The 3-year OS rate was 70.0%, and the 5-year OS rate was 50.0% (Fig. 2a). The 3-year PFS rate was 55.0%, and the 5-year PFS rate was 50.0% (Fig. 2b). At the time of analysis (September 2023), nine patients were alive without recurrence; however, nine patients had died as a result of recurrence. The first sites of recurrence were the peritoneum (*n* = 4), lung (*n* = 1), liver (*n* = 1), brain (*n* = 1), skin (*n* = 1), and distant lymph nodes (*n* = 1). The remaining two patients died of other diseases (pneumonia), 57 and 59 months after surgery, respectively. No late AEs or treatment-related deaths due to radiation were observed in any patient during the follow-up period.

## Discussion

To improve the prognosis of resectable type 4 GC, Furukawa previously performed extended resection surgery (left upper abdominal exenteration plus the Appleby procedure).^16^ However, this extended surgery has not become common, owing to the high incidence of pancreatic fistula. Therefore, we devised an alternative treatment to extended resection surgery. This treatment is based on the hypothesis that preoperative radiation, as an alternative to extended surgery, and subsequent gastrectomy with D2 lymph node dissection, would decrease surgical complications and improve prognosis. The new strategy consisted of concurrent radiotherapy and systemic chemotherapy with S-1 plus bolus cisplatin before surgery for type 4 or large type 3 GC.

In the S-1 and cisplatin (SP) regimen of the JCOG0501 trial, S-1 was administered for 3 weeks, followed by 1 week of rest. The dosages of S-1 used in the JCOG0501 trial and our study were 420 mg/m^2^/week and 280 mg/m^2^/week, respectively. The dose of cisplatin used in the JCOG0501 trial and our study was the same at 15 mg/m^2^/week.

Regarding AEs during neoadjuvant therapy, in our study, grade 3/4 leukopenia occurred in 35% of the patients, grade 3/4 neutropenia occurred in 25%, and grade 3/4 nonhematological AEs occurred in 5%. In contrast, in the JCOG0501 trial, the use of the SP regimen resulted in grade 3/4 leukopenia (7.5%), grade 3/4 neutropenia (29%), grade 3/4 anemia (4.1%), and grade 3/4 nonhematological AEs (11.6%).^14^ The high frequency of leukopenia in our study compared with the JCOG study may be owing to the effects of radiotherapy. However, there were no treatment-related deaths during neoadjuvant CRT.

One patient did not undergo surgery owing to a grade 4 AE (hyponatremia); therefore, the operation completion rate was 95% (19/20 patients). Regarding the R0 resection rate, our study achieved an 85% (17/20 patients) R0 resection rate, which was slightly better than that in the JCOG0501 NAC group (80.6%).^17^

Notably, in this study, two (10.5%) cases of P1 with peritoneal cytology positive for carcinoma cells (CY1) were observed after neoadjuvant CRT. This finding may have resulted from the inadequate diagnostic accuracy of laparoscopic examination. Staging laparoscopy plays an important role in the detection of peritoneal metastasis that is undetectable radiologically. However, the false-negative rate of laparoscopic screening ranges from 10 to 17.2% for large type 3 and type 4 GC.^18–20^ Therefore, in our study, P1 or CY1 might have been latent at the time of initial staging laparoscopy.

Recently, the usefulness of the cell block technique has been reported, ^21^ and this test is covered by insurance in Japan. Therefore, the cell block technique may increase the accuracy of peritoneal cytological diagnosis in the future.

The degree of toxicity of neoadjuvant therapy is a critical problem because of its potential adverse effects on operative morbidity and operative mortality. In our study, the postoperative morbidity rate (grade ≥ 3) was 21%. In comparison, the JCOG0501 trial reported a 7.9% complication rate (grade ≥ 3) after standard D2 gastrectomy in the NAC group.^17^ The higher complication rate in our study compared with the JCOG0501 trial may be owing to tissue edema and scarring caused by radiation exposure,^22,23^ which made the surgery more difficult. Additionally, the frequency of pancreatic fistula in our study was only 5.3% compared with the 30% reported by Furukawa et al.^16^ The reduced frequency of pancreatic fistula may be owing to fibrosis of the pancreatic tissue caused by radiation.^24^ Notably, no treatment-related deaths were observed in this study.

All 17 patients who underwent R0 resection received adjuvant chemotherapy with S-1. Therefore, the treatment completion rate was 85% (17/20 patients). In comparison, the treatment completion rate in the JCOG0501 NAC group was only 47% (71/151 patients).^7^ Our treatment strategy using neoadjuvant CRT was considered safe on the basis of the high treatment completion rate.

Regarding therapeutic efficacy, our study achieved a pathological complete response rate of 10.5%, which was better than that of the JCOG0501 trial (2%).^7^

It is assumed that the increased pathological complete response rate in this study was associated with the addition of concurrent radiation therapy. Additionally, our results demonstrated a high pathological response rate of 100%, which is higher than that in the JCOG0501 trial at only 51%.^7^ Thus, our neoadjuvant CRT showed better therapeutic efficacy compared with the SP regimen in the JCOG0501trial. This result might be owing to the strong local control effect of radiation therapy. Tomasello et al. reported a histological response associated with improved survival in patients with GC who received neoadjuvant treatment.^25^

Regarding the long-term prognosis, the 3-year OS and PFS in the JCOG0501 trial NAC group were 60.9% and 47.7%, respectively.^7^ Although our study was a phase II trial, the 3-year OS and PFS were 70.0% and 55.0%, respectively, and the 5-year OS and PFS were also both 50.0%. In this study, four cases of peritoneal dissemination recurrence and five cases of distant organ recurrence were observed; however, there were no local recurrences within the irradiated areas, and furthermore, no radiation-induced late AEs occurred. Neoadjuvant CRT might provide a favorable prognosis for patients with type 4 or large type 3 GC.

Previous studies have confirmed a survival benefit of neoadjuvant CRT for esophageal and gastroesophageal junction cancers.^26–29^ In contrast, studies reporting the efficacy of neoadjuvant CRT for locally advanced GC are rare.^30–32^

The TOPGEAR trial reported that the addition of preoperative CRT to perioperative chemotherapy did not improve OS among patients with resectable GC and gastroesophageal junction cancers.^33^ However, 43% of participants in that study had histopathologic grade of G3 or G4 tumors, whereas 85% (17/20) of participants in our study had tumors equivalent to G3 or G4, suggesting differences in the biological behavior between the tumors in these two studies. Thus, the patient cohorts differ markedly between the two studies, making it invalid to simply compare their results.

Additionally, the CRITICS-II trial to compare the role of preoperative CRT with that of preoperative or perioperative chemotherapy alone using contemporary chemotherapy regimens in patients with resectable GC is underway.^34^ The results of this study are not yet available.

To the best of our knowledge, ours is the first report on the utility of neoadjuvant CRT for clinically resectable type 4 or large type 3 GC.

Because the JCOG0501 trial failed to demonstrate a survival advantage of neoadjuvant chemotherapy with a doublet regimen of S-1 plus cisplatin,^7^ the JCOG2204 trial is currently underway, its aim being to assess the efficacy of a triple regimen of FLOT (5-fluorouracil/oxaliplatin/docetaxel) or DOS (docetaxel/oxaliplatin/S-1) as neoadjuvant chemotherapy for patients with clinically resectable type 4 and large type3 GC.^35^ Comparison of our results with those of the JCOG2204 trial may clarify whether a CRT or a triplet regimen is more effective as neoadjuvant therapy in this patient population.

Furthermore, the NOBEL trial reported that additional immunotherapy with nivolumab after radical CRT is safe and improves CR in patients with esophageal cancer.^36^ The addition of immunotherapy to neoadjuvant CRT in the treatment of GC may further improve these patients’ prognosis.

Although the present study was originally planned to involve 25 patients, patient enrollment was delayed and then terminated before the projected number of patients was achieved. The eligibility criteria for this study made the recruitment difficult because the incidence of P1 or CY1 is relatively high for type 4 and large type 3 GC.

In addition, some practitioners are prejudiced against radiotherapy, believing that it is ineffective against GC and that subsequent surgery would be difficult. These beliefs may have hampered recruitment of participants.

Recently, the incidence of GC has declined owing to the widespread eradication of *Helicobacter pylori*.^37^ For better recruitment for a future clinical trial, treatment of GC may have to be consolidated in a smaller number of centers.

In conclusion, our treatment regimen with neoadjuvant CRT for clinically resectable type 4 and large type 3 GC patients is feasible and effective. This regimen should be evaluated further in a randomized phase III trial.
